# The First Record of a Trans-Oceanic Sister-Group Relationship between Obligate Vertebrate Troglobites

**DOI:** 10.1371/journal.pone.0044083

**Published:** 2012-08-28

**Authors:** Prosanta Chakrabarty, Matthew P. Davis, John S. Sparks

**Affiliations:** 1 Louisiana State University, Museum of Natural Science, Department of Biological Sciences, Baton Rouge, Louisiana, United States of America; 2 The Field Museum, Department of Zoology, Chicago, Illinois, United States of America; 3 American Museum of Natural History, Department of Ichthyology, Division of Vertebrate Zoology, New York, New York, United States of America; Ecole Normale Supérieure de Lyon, France

## Abstract

We show using the most complete phylogeny of one of the most species-rich orders of vertebrates (Gobiiformes), and calibrations from the rich fossil record of teleost fishes, that the genus *Typhleotris*, endemic to subterranean karst habitats in southwestern Madagascar, is the sister group to *Milyeringa*, endemic to similar subterranean systems in northwestern Australia. Both groups are eyeless, and our phylogenetic and biogeographic results show that these obligate cave fishes now found on opposite ends of the Indian Ocean (separated by nearly 7,000 km) are each others closest relatives and owe their origins to the break up of the southern supercontinent, Gondwana, at the end of the Cretaceous period. Trans-oceanic sister-group relationships are otherwise unknown between blind, cave-adapted vertebrates and our results provide an extraordinary case of Gondwanan vicariance.

## Introduction

Due to their limited long-distance dispersal capabilities, freshwater fishes provide critical evidence for revealing ancient biogeographic patterns [1]–[3] and those fishes that are also blind, obligate cave-dwellers represent some of the least vagile organisms on Earth [4]–[6]. Narrow endemicity in endogean organisms is due not only to their lack of sight and pigment, but also to unique physiologies and highly specialized ecological requirements [7]. Here we examine several lineages of cave-dwelling gobies residing on opposite sides of the Indian Ocean.

**Figure 1:**
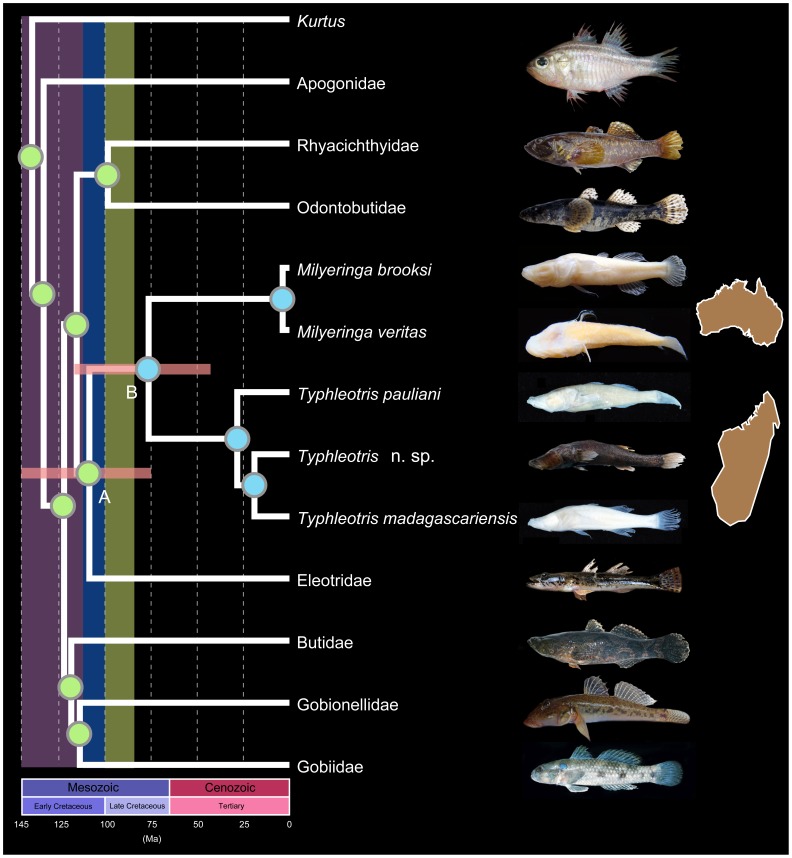
Evolutionary relationships and divergence times of Gobiiformes. Red horizontal bars represent 95% interval of potential estimated divergence times for that clade. Vertical bars represent hypothesised timing of key geologic events based on geophysical data [27]–[29], [33]–[34] including: intact Eastern Gondwana landmass (Antarctica, Australia, Indo-Madagascar) in purple; separation of Indo-Madagascar from Antarctic landmass in blue; and separation of Madagascar from India in green. Circles indicate likelihood-based ancestral character reconstructions for presence of functional eyes (green), or the lack of functional eyes, i.e., blindness (blue).

Caves and other endogean systems provide habitat to highly endemic and often bizarre organismal communities, including many ‘relict’ species, the so-called “wrecks of ancient life” of Darwin [6], [8]. The endemic northwestern Australian genus *Milyeringa* and the endemic southwestern Malagasy genus *Typhleotris* are small (<100 mm) robust fishes with a sleeper goby (Eleotridae) like morphology, except in lacking eyes and pigment (with one pigmented exception, which is also the only known darkly pigmented blind subterranean fish; Figure 1, *Typhleotris* n. sp. [9]). Although they lack functional eyes, these fishes possess elongated shovel-like snouts that are covered in neuromasts. There are five species known from these genera, three species of *Typhleotris* (one undescribed) and two species of *Milyeringa*. All are eyeless subterranean dwellers, with very restricted distributions within isolated cave-bearing limestone (karst) formations of Australia (Cape Range Peninsula) and Madagascar (Mahafaly Plateau).

Aquatic troglobites, more specifically known as stygobites, have been shown to be wider ranging in general than non-aquatic troglobites; however, this phenomenon has been demonstrated only on a very fine geographic scale for vertebrates [4]. A major issue plaguing our understanding regarding the evolution of cave animals has been a lack of basic information regarding the assembly of these biotas, including mechanisms of speciation and phylogenetic origin [6]. Despite limitations imposed by a high degree of morphological convergence and ‘regressive’ traits in many cave animals, molecular phylogenetic techniques are providing new insights [10]. Moreover, resolving the evolutionary relationships of subterranean lineages is critical not only for gaining insight into historical biogeography, but also the evolutionary processes that have contributed to these diverse and bizarre endogean biotas [11]–[14].

Here we use molecular phylogenetic methods to examine a potential trans-oceanic sister-group relationship between obligate cave-dwelling gobies. Our temporal phylogeny, based on multiple fossil calibrations, is currently the most taxonomically comprehensive hypothesis of goby relationships. The analysis utilizes four mitochondrial markers to resolve the relationships and ages of these stygobitic taxa. Some evidence has suggested that mitochondrial markers may not be ideal for dating a potentially ancient group [15]; although that study did not include multiple fossil calibrations as ours does. Mitochondrial data has been used extensively in studies that have investigated the evolutionary relationships of fishes [16]–[17], including estimations of temporal divergence [18]–[19]. Further, our current understanding of gobiiform relationships builds largely on studies that used mitochondrial data [20]–[22].

Gobies are one of the most diverse, widespread, and species-rich lineages of vertebrates and include both marine and freshwater taxa [22]. Blindness and reduced eyes have evolved rarely in fishes and in gobies in particular (which contains more than 2200 species). Besides, *Typhleotris* and *Milyeringa*, the only other truly blind gobies are additional cave species: *Glossogobius ankaranensis* (member of Gobiidae, known from caves in Madagascar and sampled for the first time in this phylogenetic study), *Caecogobius cryptophthalmus* (member of Gobiidae, known only from a few specimens from caves in the Philippines), *Oxyeleotris caeca* (member of Eleotridae, known from a few specimens from caves in Papua New Guinea), *Luciogobius pallidus*, *L.albus* and *Typhlogobius californiensis* (members of Gobiidae, marine or brackish species from seaside caves) [23]–[24]. Reduced eyes are also known from fossorial goby species such as *Traupauchen,* but these species are not blind or cave adapted. In general, the loss or reduction of eyes is rare in fishes [25], occurring in less than 1% of described species (150 species of 28,000). Blindfishes are only known from 20 families (out of more than 500 fish families); therefore, independent loss of eyes and a transition to a sytogobitic life style is a rare event [26]. In our study we examine several cave-adapted and eyeless members of the goby lineage (including species of *Typhleotris*, *Milyeringa*, *Glossogobius* and *Typhlogobius*) to examine the relationships of these fishes and to examine the history of their blindness and troglodytic life-style.

## Results and Discussion

**Figure 2:**
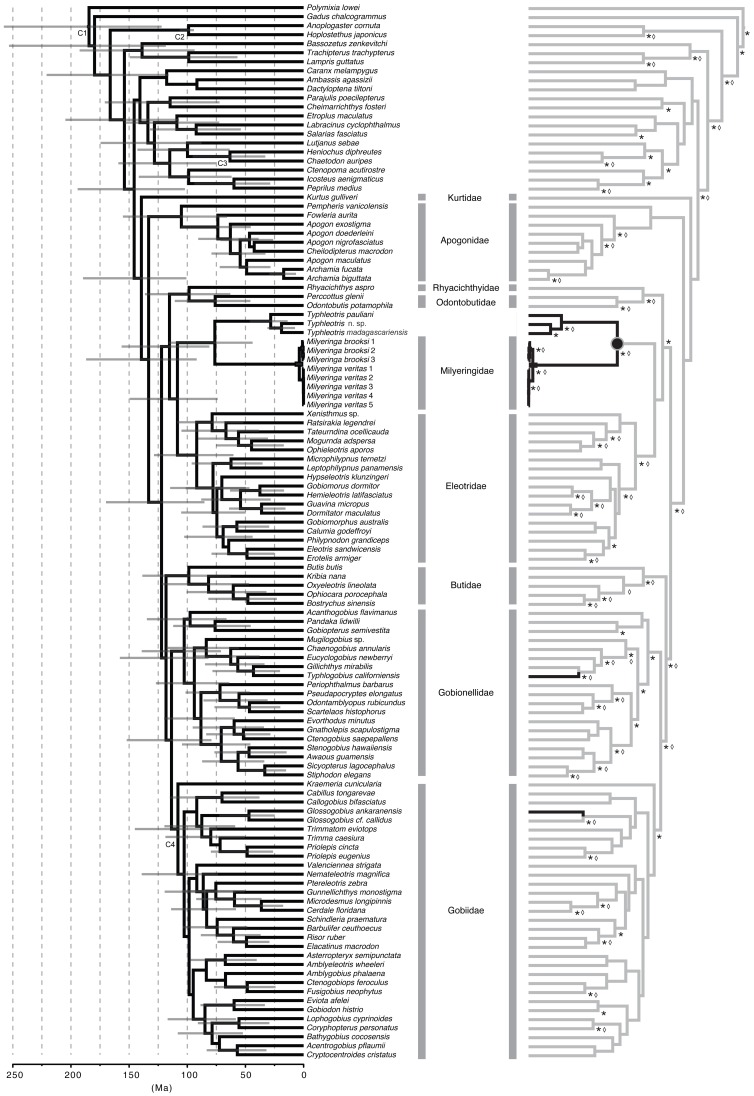
Evolutionary relationships and estimated divergence times for members of the order Gobiiformes. Grey horizontal bars represent 95% interval of potential divergence times. Phylogeny on right indicates likelihood-based ancestral character reconstructions for presence of functional eyes (grey lines), and functionally blind (black lines). An *indicates posterior probability support for node in the Bayesian analysis of greater than 95%, whereas a diamond indicates a bootstrap greater than 60% for the likelihood reconstruction.

The recovered phylogenetic hypothesis represents both the most thorough sampling of gobiiform fishes to-date, as well as the only time-calibrated tree for one of the most species-rich orders of vertebrates. Most notably, our phylogenetic analyses recovered a sister group relationship between freshwater gobies of the genus *Typhleotris*, endemic to subterranean karst habitats in southwestern Madagascar and the genus *Milyeringa*, endemic to similar subterranean systems in northwestern Australia (Figure 1, 2). Although morphologically similar externally, these taxa had not previously been demonstrated to be closely related. Our temporal phylogeny, which includes calibrations from the rich fossil record of teleost fishes, recovers an Early Cretaceous age for gobioid fishes that corroborates recent geological and geophysical data. These Earth history data indicate the last subaerial connection between the Antarctica/Australia block and the Indo-Madagascar landmasses occurred during the Early Cretaceous [27]. Our results present a compelling example of an ancient vicariant pattern, given the limitations for long-distance dispersal of obligate subterranean lineages and the contemporary trans-oceanic distributions of these two lineages.

**Figure 3:**
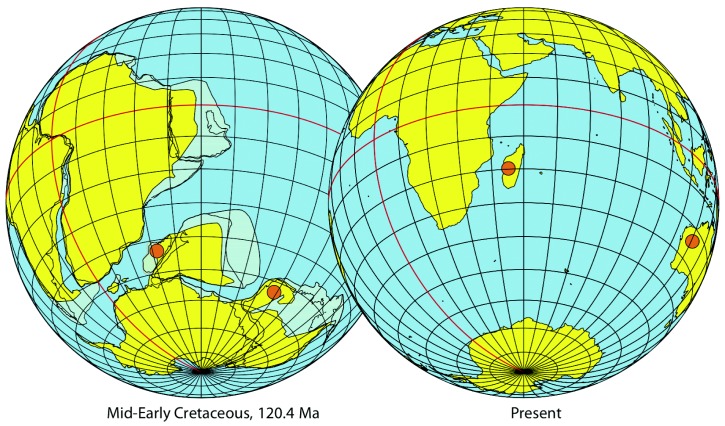
Maps showing the Gondwana continents in the mid-Early Cretaceous (left) and at present (right), with orange dots showing the current localities of *Typhleotris* (Madagascar) and *Milyeringa* (Australia).

Geological and geophysical data for the regions that once formed eastern Gondwana indicates that Australia maintained a direct connection to Antarctica throughout the Cretaceous (145-65 Ma; Figure 3) [27]–[29]. However, there is considerable debate surrounding the location of the latest point of connectivity of the Indo-Madagascar landmass to Antarctica, as well as the timing of rifting leading to their subaerial isolation. A number of studies have suggested that Antarctica remained connected to Indo-Madagascar through causeways until the Late Cretaceous (≈80 Ma), either via the Gunnerus Ridge [30]–[31], or the Kerguelen Plateau [32]. However, using current geophysical reconstructions to extrapolate the fit of these landmasses at the end of the Cretaceous provides little direct evidence for the existence of continuous causeways that would permit terrestrial biotic interchange between the landmasses [27], particularly in light of magnetic anomaly dating limitations imposed by the Cretaceous Quiet Zone (KQZ) [33]–[34]. In the absence of Late Cretaceous causeways, current geologic evidence indicates that the last terrestrial connection between Indo-Madagascar and Antarctica persisted until the mid-Aptian stage of the Early Cretaceous (≈115 Ma) [27]–[29], [33]–[34].

Our hypothesis of evolutionary relationships among major lineages of gobiiform fishes based on likelihood and Bayesian reconstructions of nucleotide characters (Figure 1, 2) recovered *Milyeringa* and *Typhleotris* as sister lineages with high support (>100% posterior probability; 73% bootstrap support; Figure 2). This lineage of cave fishes was recovered as the sister group of the very diverse and species rich Eleotridae (sleeper gobies; 130 species [35]). Eleotrids have a widespread distribution, and occur in both marine and freshwater environments throughout the Indo-Pacific and Neotropics. *Milyeringa* and *Typhleotris* have previously been considered to be members of Eleotridae [36]. Within the suborder Gobioidei [21], we recovered a clade comprising four lineages, with the predominantly Asian lineages Rhyacichthyidae + Odontobutidae recovered as the sister taxon to a *Milyeringa* + *Typhleotris* + Eleotridae clade (Figure 1, A). The clade including Rhyacichthyidae, Odontobutidae, *Milyeringa*, *Typhleotris*, and Eleotridae was recovered as the sister taxon to all remaining Gobioidei families (i.e., Butidae, Gobionellidae, Gobiidae) as shown in Figures 1 and 2. Estimates of divergence times calculated using Bayesian approaches with multiple teleost fossil calibrations recover ages for these lineages that are congruent with the Early through Late Cretaceous fragmentation of Eastern Gondwana (Figure 1–2, Table 1).

**Table 1:** Estimated divergence times of gobioid lineages.

Clade	Mean Age (95% HPD)
Gobiiformes	140 Ma (190–101 Ma)
Gobioidei	122 Ma (170–85 Ma)
Rhyacichthyidae + Odontobutidae	98 Ma (136–63 Ma)
*Milyeringa* + *Typhleotris* + Eleotridae (A)	109 Ma (150–74 Ma)
*Milyeringa* + *Typhleotris* (B)	77 Ma (116–44 Ma)
*Typhleotris*	28 Ma (46–14 Ma)
*Milyeringa*	4 Ma (7–1.5 Ma)
Eleotridae	92 Ma (129–60 Ma)
Butidae	99 Ma (139–62 Ma)
Gobionellidae	103 Ma (139–71 Ma)
Gobiidae	108 Ma (145–74 Ma)

Divergence times correspond to those depicted in Figures 1 and 2. Ages are expressed in millions of years ago (Ma), with the mean age and the 95% higher posterior densities of potential divergence times (HPD). Letter in parentheses corresponds to the clade in Figure 1.

The sister-group relationship between *Milyeringa* and *Typhleotris* represents an interesting example of a possible relict Gondwanan lineage, potentially isolated in subterranean karst habitats since the Mesozoic breakup of the southern supercontinent. Geophysical reconstructions of Gondwana do not include a scenario in which Australia and Madagascar directly abut each other, therefore it is likely that the common ancestor of the clade comprising *Milyeringa* + *Typhleotris* and/or the clade comprising *Milyeringa* + *Typhleotris* + Eleotridae clade was distributed throughout Eastern Gondwana (particularly India) during the Early Cretaceous because the age of this lineage corresponds to a time when this supercontinent was intact (Figure 1, A to B). The exposed karst environments of Madagascar and Australia that individuals of *Typhleotris* and *Milyeringa* currently inhabit are also of similar geologic age (Eocene) and composition [37]–[38]. These karst window habitats are likely younger than their deeper subterranean connections, but this relatively young age suggests that this lineage had a much broader ancient distribution across the greater Eastern Gondwanan region. Additional diversity may exist in Madagascar and Australia, and rigorous sampling efforts by the authors in the known karst habitats of both countries have yielded new species [9], [10]; however, given the sampling efforts to date it is unlikely that these lineages would be profoundly more diverse than is currently known on these two landmasses.

Few organisms have more limited long-distance dispersal capabilities than troglobites, which are more or less tied to their specific subterranean habitat [6]. The absence of eyes and pigment makes troglobites easy targets for predators when exposed outside of their isolated subterranean habitats, that often lack these predators. The lack of pigmentation also presents physiological limitations related to length of exposure to UV radiation from sunlight. The absence of these traits are of no consequence in their dark isolated subterranean environments; however, features such as protective pigmentation and sight are likely required for successful dispersal outside of those hypogean habitats. Notable exceptions do exist among marine cave taxa that are similarly vulnerable: Blind anchialine invertebrate stygiobionts, such as members of Remipedia, have a global distribution and may be capable of long-distance dispersal [39]. However, dispersal between landlocked subterranean habitats are not well studied among vertebrates and disjunctions even within a single landmass are extremely rare; a transoceanic sister relationships as the one discussed here are otherwise unknown. A likelihood ancestral character reconstruction of the loss of functional eyes across the suborder Gobioidei which includes other blind species (e.g. *Glossogobius, Typhlogobius*) indicates that the common ancestor of the *Milyeringa* + *Typhleotris* clade was most likely blind (Figure 1–2), whereas the common ancestor of the *Milyeringa* + *Typhleotris* + Eleotridae clade most likely had functional eyes. (A single blind species of Eleotridae, only known from the types, *Oxyeleotris caeca,* was not included in this study because no samples are available). Consequently, any post-Gondwanan breakup dispersal hypothesis would potentially require interconnected subterranean habitats connecting the former Eastern Gondwanan landmasses to account for the disjunct distribution. This dispersal scenario is highly unlikely, particularly given the lack of geological and geophysical evidence for the existence of any such subterranean causeways during the Late Cretaceous [27]–[29], [33]–[34].

Recent long-distance dispersal or molecular sequence convergence scenarios are far less likely explanations than vicariance of widespread ancestral populations that were present in Eastern Gondwana. Issues of long-branch attraction have been demonstrated to impact parsimony analyses more severely than model-based approaches (maximum likelihood and Bayesian methodologies), where an artificial relationship resulting from long-branch attraction is less likely to be recovered [40]–[41]. We did not remove third codon positions from our analyses because positions that may have increased saturation have been demonstrated to provide additional and critical phylogenetic signal [42]. Furthermore, there are no observable indications of exceedingly long branches among taxa in our analyses and there is no evidence to suggest that long-branch attraction is causing the inference of any spurious phylogenetic hypotheses in this study. All previous large-scale molecular phylogenies of gobies are based on mitochondrial loci [20]–[22], which restricts the type of genetic data we use here to only mitochondrial information. Future work using nuclear DNA is planned. However, the use of mitochondrial loci currently allows for a breadth of taxonomic sampling within gobiiform fishes that allow us to investigate whether cavefishes in *Milyeringa* and *Typhleotris* are closely related. The sister-group relationship between these two genera is well resolved and strongly supported (Fig. 2).

It is possible that the ancestor of the *Milyeringa* + *Typhleotris* clade may have exhibited a higher salinity tolerance than extant populations, sufficient for entering a marine environment (some populations of *Milyeringa veritas* are known from brackish habitats [36]); however, juveniles and/or larvae of *Milyeringa* or *Typhleotris* have never been recovered in marine habitats, and *Typhleotris* is not salt tolerant. Given the Cretaceous age of the *Milyeringa* + *Typhleotris* clade, it is also possible that extinction has had an impact on this clade, with the extant subterranean lineages persisting as relictual populations from a formerly wider distribution across Gondwana. This distribution may have included non-cave dwelling species of *Milyeringa* and/or *Typhleotris*, however there is no evidence of non-subterranean members of the *Milyeringa* + *Typhleotris* lineage either from the fossil record or extant species.

Our character state reconstructions of eye reduction and loss support a single loss of functional eyes in the common ancestor of the *Milyeringa* + *Typhleotris* clade. Adult and larval forms of *Milyeringa* lack eyes (larvae of *Typhleotris* have not been observed, but adults and juveniles are eyeless). Although some eleotrids (the sister lineage to the *Milyeringa* + *Typhleotris* clade) are freshwater inhabitants as adults and disperse in the marine realm as juveniles, no individuals of *Milyeringa* or *Typhleotris* have ever been observed outside of their restricted karst habitat. However, the sister relationship between these obligate cave dwellers and the widely distributed Eleotrids may lend credence to a greater dispersal ability in *Milyeringa* or *Typhleotris* taxa, or a wider distribution of their shared ancestor. However, there is currently no evidence of for widespread dispersal capabilities in taxa within *Milyeringa* and *Typhleotris*.

Our divergence time estimates of Gobiiformes, calibrated using the fossil record of teleosts, are congruent with the existence of a widespread Early Cretaceous ancestor throughout Eastern Gondwana. At present, several widely-distributed freshwater fish assemblages exhibit phylogenetic patterns of relationship that are congruent with the temporal sequence of the breakup of Western and Eastern Gondwana during the Jurassic and Cretaceous, including cichlids, melanotaeniid rainbowfishes, and aplocheilid killifishes [3]. Although a sister-group relationship between stygobites endemic to similar karst habitats on opposite ends of the Indian Ocean might seem highly unlikely, our results indicate that the evolutionary timing of divergence for gobioid fishes is consistent with a Gondwanan vicariance hypothesis. Given these data, this Gondwanan vicariance hypothesis is the simplest explanation for the incredible disjunct distribution of this lineage of Malagasy and Australian obligate cave fishes.

## Materials and Methods

Four loci (4846 bp from ND1, ND2, cytB, and COI) were sequenced for several populations across the range of *Milyeringa* and *Typhleotris* and include all known species, including undescribed forms (one representative population per species was included in the final analysis). Loci were selected to permit incorporation of the largest possible taxon sampling of Gobiiformes by adding to the datasets of Thacker and others [16], [20]–[21] and our tree now represents the most taxonomically robust dataset for gobiiforms. Outgroups included a breadth of acanthomorph lineages (Figure 2). Each gene was assigned a separate model of nucleotide substitution based on the Akaike information criteria (AIC) performed in jMODELTEST 0.1.1 [43], including HKY+G (COI), GTR+G (ND1), and GTR+I+G (ND2, cytB) and sequences were aligned with MAFFT [44] using default parameters. Novel sequences were submitted to GenBank (Table 2) and the final alignment is available in Dryad (http://datadryad.org/).

**Table 2:** GenBank accession numbers for molecular samples used in phylogenetic analyses.

GenBank #	Gene	Taxon	AMNH Cat #
**JQ619660**	**CytB**	***Typhleotris*** ** new sp.**	**245601**
JQ619661	CytB	*Typhleotris madagascariensis*	245609
JQ619662	CytB	*Typhleotris pauliani*	245649
JQ619663	CytB	*Glossogobius ankaranensis*	245682
JQ619664	CytB	*Glossogobius callidus*	245685
**JQ619665**	**COI**	***Typhleotris*** ** new sp**	**245601**
JQ619666	COI	*Typhleotris madagascariensis*	245609
JQ619667	COI	*Typhleotris pauliani*	245649
JQ619668	COI	*Glossogobius ankaranensis*	245682
JQ619669	COI	*Glossogobius callidus*	245685
**JQ619670**	**ND1**	***Typhleotris*** ** new sp.**	**245601**
JQ619671	ND1	*Typhleotris madagascariensis*	245609
JQ619672	ND1	*Typhleotris pauliani*	245649
JQ619673	ND1	*Glossogobius ankaranensis*	245682
JQ619674	ND1	*Glossogobius callidus*	245685

American Museum of Natural History (AMNH) catalog number is listed in the last column. “*Typhleotris* new sp.” refers to a new species being described [9]. Genetic sequences from the holotype of the new species are hologenetypes, following the nomenclature of Chakrabarty [53] and are in bold.

Topologies reconstructions and relative divergence times were estimated simultaneously using BEAST v.1.6.1 [45] with an XML template generated from BEAUTI v1.6.1 and results visualized in TRACER v.1.5 [46]. Each gene was assigned a separate partition based on the results from jMODELTEST test. Four independent runs were performed with 50 million generations each, with a burnin of 10 million generations for each analysis. Trees were sampled every 10,000 iterations, for a total of 20,000 trees (16,000 post-burnin). The effective sample size of all parameters converged on a stationary distribution. A 50% maximum clade credibility (mean heights) tree was generated from the posterior tree distribution (Figures 1, 2).

A maximum likelihood topology reconstruction was performed in GARLI 2.0 [47] with each gene assigned a separate partition. The likelihood analysis was replicated ten times, and topologies were identical to the mean tree recovered in the Bayesian analysis (Figure 2). Likelihood-based ancestral character state reconstruction was performed in Mesquite 2.7 [48] (Figure 1, 2).

Fossil calibrations were assigned a lognormal prior, with hard minimum ages based on the oldest known fossil of the respective lineages. A conservative soft upper bound was set to 150 Ma for all calibrations, the age of the oldest known fossil euteleost, †*Leptolepides sprattiformis* [49]. Acanthomorpha (C1): A minimum age of 94 Ma was used based on fossil taxa from the extant stem acanthomorph lineage *Polymixia* [50]. Beryciformes (C2): A minimum age of 94 Ma was used based on the fossil taxa †*Hoplopteryx simus* and †*Hoplopteryx lewesiensis* known from Middle–Upper Cenomanian deposits [50]. Chaetodontidae (C3): The minimum age of 30 Ma was assigned based on the oldest fossil representative of the family †Chaetodonidae cf. *Chaetodon* known from Rupelian deposits [51]. Gobiidae (C4): Minimum age of the family Gobiidae was established based on fossils identified in Miller [52] as belonging to this family with an Eocene age of 33.9 Ma.
